# Constipation in multiple system atrophy: a pilot study in Chinese patients

**DOI:** 10.3389/fneur.2023.1202279

**Published:** 2023-06-09

**Authors:** Yalan Chen, Hongyan Huang, Peng Zhang, Yanming Xu, Yangmei Chen

**Affiliations:** ^1^Department of Neurology, The Second Affiliated Hospital of Chongqing Medical University, Chongqing, China; ^2^Department of Neurology, West China Hospital, Sichuan University, Chengdu, Sichuan, China

**Keywords:** multiple system atrophy, constipation, prevalence, non-motor symptom, ROME III criteria

## Abstract

**Objective:**

This study aimed to investigate the prevalence and clinical characteristics of subjective constipation in Chinese patients with multiple system atrophy (MSA), as well as the timing of constipation onset relative to the occurrence of motor symptoms.

**Methods:**

A total of 200 patients who were consecutively admitted to two large Chinese hospitals from February 2016 to June 2021 and subsequently diagnosed with probable MSA were enrolled in this cross-sectional study. Demographic and constipation-related clinical data were collected, and motor and non-motor symptoms were assessed using various scales and questionnaires. Subjective constipation was defined using ROME III criteria.

**Results:**

The frequency of constipation was 53.5, 59.7, and 39.3% in MSA, MSA with predominately parkinsonism (MSA-P), and MSA with predominately cerebellar ataxia (MSA-C), respectively. MSA-P subtype and high total UMSARS scores were associated with constipation in MSA. Similarly, the high total UMSARS scores were associated with constipation in MSA-P and MSA-C patients. Among the 107 patients with constipation, 59.8% began experiencing it before the onset of motor symptoms, and the interval between constipation and occurrence of motor symptoms was significantly longer in these patients than in those who experienced constipation after onset of motor symptoms.

**Conclusion:**

Constipation is a highly prevalent non-motor symptom in MSA and more often occurs before the onset of motor symptoms. The results of this study may help guide future research into MSA pathogenesis in its earliest stages.

## Introduction

Multiple system atrophy (MSA) is a sporadic, rapidly progressive neurodegenerative disorder characterized by autonomic dysfunction, including orthostatic hypotension, bladder dysfunction, gastrointestinal dysfunction, and sexual dysfunction, along with parkinsonism or cerebellar ataxia (1). Constipation has also been observed in MSA patients, and it appears to emerge earlier than in patients with Parkinson’s disease (PD) (2). Constipation is considered an early pre-motor manifestation of PD, occurring in 8–70% of patients (3). The prevalence of constipation and its timing relative to the onset of motor symptoms in MSA are unclear, reflecting the low incidence of MSA.

Here we investigated these questions in Chinese patients with MSA. Our findings may help clarify how MSA arises and progresses in its earliest stages.

## Methods

### Participants

A total of 200 patients who were consecutively admitted to West China Hospital of Sichuan University and The Second Affiliated Hospital of Chongqing Medical University from February 2016 to June 2021 and subsequently diagnosed with probable MSA (4) were enrolled in this cross-sectional study. Patients with other neurodegenerative disorders, prior gastro-intestinal surgery, or any other identifiable cause of constipation were excluded.

All enrolled patients were interviewed in person, and their socio-demographic and clinical data were collected. The study was approved by the Ethics Committee of West China Hospital of Sichuan University, and all patients provided written informed consent.

### Clinical assessments

Motor and non-motor symptoms were evaluated in all patients using the Unified MSA Rating Scale (UMSARS) (5), Non-motor Symptoms Scale (NMSS), Mini-mental State Examination (MMSE) (6), Hamilton Anxiety Rating Scale (HAMA), Hamilton Depression Rating Scale (HAMD), and Pittsburgh Sleep Quality Index (PSQI) (7).

Constipation, one of the most frequent non-motor symptoms of gastrointestinal dysfunction in the autonomic system, can be defined based on subjectively perceived symptoms or on objectively measured intestinal transit time. In the present study, Constipation was defined based on the ROME III criteria as the presence of two or more of the following symptoms (8): straining to defecate, lumpy/hard stool, sensation of incomplete evacuation, sensation of anorectal obstruction, manual maneuvering, and fewer than three bowel movements per week. These symptoms had to be present during at least 25% of defecations during at least 3 months after symptom onset, which had to be at least 6 months before the present study. Patients with constipation were asked to report the time of constipation onset relative to the onset of motor symptoms.

### Statistical analysis

Continuous variables were reported as mean and standard deviation, and categorical variables as counts and percentages. Student’s *t* test was used to compare continuous variables between patients with and without constipation in the MSA group, MSA-P subgroup, and MSA-C subgroup. The chi-squared or Fisher’s exact tests was used to compare the categorical variables between different groups. Multivariate binary logistic regression models were used to determine the factors related to constipation. Variables associated with constipation at *p* values <0.05 or possibly associated with constipation based on clinical judgment in the univariate analysis were included in the multivariate binary logistic regression model. Results were expressed as odds ratios (ORs) and 95% confidence intervals (95% CIs). All statistical analyses were conducted using SPSS 19 (IBM, Chicago, IL, United States), and differences associated with *p* < 0.05 were considered significant.

## Results

Demographic characteristics of MSA, MSA-P, and MSA-C patients with and without constipation are shown in Table 1. A total of 200 patients (77 female, 38.5%) with probable, idiopathic MSA were enrolled in the study, of whom 107 (53.5%) had constipation. The mean age of all patients was 63.88 ± 10.58 years, and they had MSA for a mean of 2.28 ± 1.78 years. The prevalence of constipation was 59.7% among the 139 patients with the parkinsonian subtype of MSA (MSA-P) and 39.3% among the 61 patients with the cerebellar subtype (MSA-C).

**Table 1:** Demographic characteristics of MSA, MSA-P, and MSA-C patients with and without constipation.

Characteristic	Total	MSA			MSA-P			MSA-C		
		Constipation	No constipation	*p*	Constipation	No constipation	*p*	Constipation	No constipation	*p*
*N*	200	107	93		83	56		24	37	
Female	77 (38.5)	42 (39.3)	35 (37.6)	0.815	31 (37.3)	20 (35.7)	0.844	11 (45.8)	15 (40.5)	0.683
Age (years)	63.88 ± 10.58	65.58 ± 10.76	61.91 ± 10.06	0.014^*^	66.94 ± 10.43	62.43 ± 10.17	0.013^*^	60.88 ± 10.79	61.14 ± 9.99	0.924
Age at MSA onset (years)	61.53 ± 10.26	63.08 ± 10.42	59.76 ± 9.84	0.022^*^	64.48 ± 10.07	60.20 ± 10.03	0.015^*^	58.21 ± 10.35	59.08 ± 9.63	0.738
MSA duration (years)	2.28 ± 1.78	2.48 ± 1.87	2.06 ± 1.65	0.094	2.52 ± 1.97	2.17 ± 1.72	0.285	2.36 ± 1.52	1.89 ± 1.54	0.251
MSA-P	139 (69.5)	83 (77.6)	56 (60.2)	0.008^*^	–	–	–	–	–	–
Levodopa	123 (61.5)	72 (67.3)	51 (54.8)	0.071	65 (78.3)	43 (76.8)	0.832	7 (29.2)	8 (21.6)	0.504
Dopamine agonists	27 (13.5)	16 (15.0)	11 (11.8)	0.519	13 (15.7)	11 (19.6)	0.543	3 (12.5)	0	0.029^*^
Amantadine	14 (7.0)	7 (6.5)	7 (7.5)	0.785	7 (8.4)	3 (5.4)	0.493	0	4 (10.8)	0.098
Benzhexol	7 (3.5)	2 (1.9)	5 (5.4)	0.179	2 (7.1)	4 (2.4)	0.180	0	1 (2.7)	0.421
Hypertension	44 (22.0)	25 (23.4)	19 (20.4)	0.617	20 (24.1)	14 (25.0)	0.903	5 (13.5)	5 (20.8)	0.454
Diabetes mellitus	21 (10.5)	15 (14.0)	6 (6.5)	0.082	11 (13.3)	2 (3.6)	0.055	4 (16.7)	4 (10.8)	0.512

MSA, multiple system atrophy; MSA-P, multiple system atrophy with predominately parkinsonism; MSA-C, multiple system atrophy with predominately cerebellar ataxia.

Values are *n* (%) or mean ± SD.

* Significant difference.

In the MSA group, patients with or without constipation did not differ significantly in sex distribution, concurrent diseases, medication, disease duration, or scores on the HAMD and MMSE. However, patients with constipation showed higher UMSARS scores total and separately on Parts I, II, and IV, indicating more advanced disease and greater disability. Patients with constipation were also significantly older, showed higher incidence of MSA-P subtype, and scored higher on the NMSS and HAMA (Table 2).

**Table 2:** Clinical assessments of MSA, MSA-P, and MSA-C patients with and without constipation.

Characteristic	Total	MSA			MSA-P			MSA-C		
		Constipation	No constipation	*p*	Constipation	No constipation	*p*	Constipation	No constipation	*p*
UMSARS scores										
Part I (historical review)	13.53 ± 5.86	15.25 ± 5.73	11.55 ± 5.38	<0.001^*^	15.00 ± 5.44	11.64 ± 5.54	0.001^*^	16.13 ± 6.69	11.41 ± 5.21	0.003^*^
Part II (motor examination)	17.35 ± 7.53	18.96 ± 7.41	15.48 ± 7.27	0.001^*^	18.89 ± 7.32	16.25 ± 7.59	0.042^*^	19.21 ± 7.84	14.32 ± 6.68	0.012^*^
Part IV (global disability)	1.82 ± 1.01	2.00 ± 1.09	1.60 ± 0.86	0.005^*^	1.96 ± 1.11	1.59 ± 0.89	0.037^*^	2.13 ± 1.0	1.62 ± 0.83	0.044^*^
Total (Parts I + II)	30.88 ± 12.48	34.21 ± 12.06	27.03 ± 11.89	<0.001^*^	33.89 ± 11.83	27.89 ± 12.49	0.005^*^	35.33 ± 13.03	25.73 ± 10.99	0.003^*^
HAMA score	11.71 ± 8.82	13.04 ± 8.84	10.14 ± 8.58	0.022^*^	13.29 ± 8.78	10.24 ± 8.54	0.047^*^	12.13 ± 9.19	10.00 ± 8.76	0.375
HAMD score	10.00 ± 7.50	10.39 ± 7.04	9.54 ± 8.01	0.433	10.21 ± 6.92	9.37 ± 7.94	0.516	11.04 ± 7.58	9.81 ± 8.23	0.564
NMSS score	54.79 ± 35.47	63.79 ± 35.29	44.97 ± 33.19	0.001^*^	62.27 ± 35.54	48.16 ± 31.55	0.029^*^	69.33 ± 34.79	39.39 ± 35.78	0.008^*^
MMSE score	24.76 ± 4.74	24.82 ± 4.32	24.68 ± 5.21	0.830	24.90 ± 4.30	25.14 ± 5.01	0.764	24.54 ± 4.43	23.97 ± 5.49	0.672
PSQI score	7.52 ± 4.67	8.38 ± 4.45	6.51 ± 4.74	0.006^*^	8.55 ± 4.49	6.37 ± 5.30	0.013^*^	7.78 ± 4.38	6.71 ± 3.85	0.332

MSA, multiple system atrophy; MSA-P, multiple system atrophy with predominately parkinsonism; MSA-C, multiple system atrophy with predominately cerebellar ataxia; HAMA, Hamilton anxiety rating scale; HAMD, Hamilton depression rating scale; MMSE, mini-mental state examination; NMSS, non-motor symptom scale; PSQI, Pittsburgh sleep quality index; UMSARS, unified MSA rating scale.

Values are *n* (%) or mean ± SD.

* Significant difference.

In the MSA-P subgroup, patients with constipation showed higher UMSARS scores total and separately on Parts I, II, and IV than those without constipation. They also scored higher on the NMSS and HAMA. In the MSA-C subgroup, patients with constipation showed higher UMSARS scores total and separately on Parts I, II, and IV. They also reported a higher frequency of dopamine agonists use and scored higher on the NMSS (Table 2).

To further investigate the factors associated with constipation, multivariate binary logistic regression was performed. In the MSA group, the MSA-P subtype (OR 2.077, 95% CI 1.065–4.049, *p* = 0.032) and total UMSARS score (OR 1.048, 95% CI 1.016–1.080, *p* = 0.003) were associated with constipation. In the MSA-P subgroup, the total UMSARS score (OR 1.038, 95% CI 1.005–1.073, *p* = 0.025) was associated with constipation. Similarly, the total UMSARS score (OR 1.063, 95% CI 1.011–1.119, *p* = 0.018) was associated with constipation in patients with MSA-C (Table 3).

**Table 3:** Factors associated with constipation in MSA, MSA-P, and MSA-C patients in multivariate regression model.

Variables	MSA			MSA-P			MSA-C		
	OR	95% CI	*p* value^a^	OR	95% CI	*p* value^b^	OR	95% CI	*p* value^c^
MSA-P subtype	2.077	1.065–4.049	0.032^*^	–	–	–	–	–	–
Total UMSARS score	1.048	1.016–1.080	0.003^*^	1.038	1.005–1.073	0.025^*^	1.063	1.011–1.119	0.018^*^

MSA, multiple system atrophy; MSA-P, multiple system atrophy with predominately parkinsonism; MSA-C, multiple system atrophy with predominately cerebellar ataxia; UMSARS, unified multiple system atrophy rating scale; OR, odds ratio; CI, confidence interval.

a Sex, age, MSA duration, HAMA score, MSA-P subtype, and total UMSARS score in the multivariate model.

b Sex, age, MSA duration, and total UMSARS score in the multivariate model.

c Age, dopamine agonists, and total UMSARS score in the multivariate model.

* Significant difference.

Among the 107 MSA patients with constipation, 64 (59.8%) reported that it began before the onset of motor symptoms. The groups in whom constipation preceded or followed motor symptom onset did not differ significantly in age, sex distribution, medication, age at motor symptom onset, age at constipation onset, MSA subtype distribution, or scores on the UMSARS (total or separately on Parts I, II or IV), HAMA, HAMD, MMSE, NMSS, or PSQI. However, the interval between constipation onset and motor symptom onset was significantly longer among patients who experienced constipation before motor symptoms than among patients who first experienced motor symptoms (Table 4).

**Table 4:** Comparison of MSA patients in whom constipation preceded or followed onset of motor symptoms.

Characteristic	Constipation first	Motor symptoms first	*p*
*N*	64	43	
Female	24 (37.5)	12 (28.1)	0.385
Age (years)	67.50 ± 11.01	64.75 ± 9.40	0.250
MSA-P subtype	49 (76.6)	34 (79.1)	0.913
Levodopa	43 (67.2)	28 (65.1)	0.923
Dopamine agonists	7 (10.9)	5 (11.6)	0.349
Age at motor symptom onset (years)	65.49 ± 10.71	61.65 ± 9.29	0.102
Age at constipation onset (years)	59.49 ± 11.45	63.14 ± 9.37	0.137
Interval between constipation onset and motor symptom onset (years)	6.01 ± 8.14	1.49 ± 0.98	< 0.001^*^
UMSARS scores			
Part I (historical review)	14.69 ± 5.75	15.69 ± 5.07	0.427
Part II (motor examination)	17.67 ± 7.29	20.56 ± 6.83	0.078
Part IV (global disability)	1.96 ± 1.17	2.00 ± 1.08	0.872
Total (Parts I + II)	32.35 ± 11.82	36.25 ± 10.66	0.137
HAMA score	12.60 ± 8.78	13.09 ± 7.99	0.798
HAMD score	10.26 ± 6.25	10.13 ± 7.24	0.935
NMSS score	63.51 ± 37.37	66.24 ± 19.49	0.694
MMSE score	25.35 ± 3.83	24.28 ± 4.27	0.245
PSQI score	9.11 ± 4.55	7.65 ± 3.74	0.141

MSA, multiple system atrophy; MSA-P, multiple system atrophy with predominately parkinsonism; HAMA, Hamilton anxiety rating scale; HAMD, Hamilton depression rating scale; MMSE, mini-mental state examination; NMSS, non-motor symptom scale; PSQI, Pittsburgh sleep quality index; UMSARS, unified MSA rating scale.

Values are *n* (%) or mean ± SD.

* Significant difference.

## Discussion

In recent years, pre-motor symptoms in MSA, such as constipation, have received increasing attention (9, 10). However, the low incidence of MSA has made it difficult to research the prevalence of constipation among patients, or the timing of constipation relative to the onset of motor symptoms. Here we provide evidence in a Chinese cohort that constipation can affect a substantial proportion of MSA patients, and that it more often precedes, rather than follows, the onset of motor symptoms. We further identified MSA-P subtype and total UMSARS score as independent risk factors of constipation in MSA.

A wide range of subjective criteria have been reported for diagnosing constipation in PD (3), but only the ROME criteria are accepted by gastroenterologists worldwide (8), in part because these criteria take into account colonic and anorectal symptoms. Constipation in MSA probably results from slow colonic transit, decreased phasic rectal contraction, and weak abdominal strain (11). Based on the ROME III criteria, we found that 53.5% of our patients suffered subjective constipation, similar to the 54.7% reported for Chinese patients based solely on item 21 of the NMSS (12), but much lower than the 82% reported for South Korean patients based on item 12 of the UMSARS (13). This discrepancy can be attributed to different diagnostic criteria for constipation and to demographic differences among the samples. Consistent with both of those studies (12, 13), constipation in our sample was more frequent among MSA-P patients than MSA-C patients, which may reflect that the MSA-P subtype affects the basal ganglia to a greater extent, or peripheral causes like more *α*-synuclein deposition in autonomic plexus of gut in MSA-P patients. Future availability of an *α*-synuclein imaging tracer would allow more direct testing of the interesting hypothesis.

We found that constipation in MSA was associated with older age, reflecting the association between constipation and older age in the general population (14), which has been attributed to degeneration of epithelial, muscle, and neural cells of the colon and pelvic floor. In contrast, we did not detect an association between constipation and sex, in contrast to the association between female sex and chronic constipation in the general population (15). Similarly, we did not detect an association between constipation and use of medications, in contrast to the association between constipation and use of dopamine agonists or other dopaminergic medication in individuals with PD (16, 17). Regrettably, the dose of drugs taken for MSA patients at the time of the visit were not recorded. So, we cannot calculate the levodopa daily equivalent dose. These differences between our study and the literature likely reflect the pathology of MSA.

Our results link constipation in MSA to worse motor performance and non-motor performance, specifically anxiety and sleep quality. Whether constipation is a cause or effect of worse disease progression should be explored in future work. In this regard, it may be interesting that the interval between constipation onset and motor symptom onset was significantly longer among patients who experienced constipation before motor symptoms than among patients who first experienced motor symptoms. It would be important to clarify risk factors specifically for constipation that precedes motor symptoms in MSA, which we could not examine here because of the small sample.

Our study suggests that constipation can precede motor symptoms of MSA by as many as 6 years. One possible explanation for what triggers prodromal constipation in MSA is *α*-synuclein deposition in the enteric autonomic nervous system (18), especially given that PD seems to involve such deposition in the enteric system at an early stage, after which the deposition spreads to the brain (gut-first subtype) (19, 20). Considering the similar pathological features of MSA and PD, it seems that MSA can also be divided into “brain first” vs. “gut first” like that in PD (21). This study is an important and welcome addition to the clinical subtyping literature in MSA.

Our findings should be interpreted with caution in light of several limitations. There were no age and sex-matched healthy controls to compare the onset of constipation. We did not investigate the effects of diet, water intake, or exercise on the occurrence of constipation, and we relied on patients’ recall to analyze the timing of constipation and motor symptom onset. We relied on patients’ self-report of symptoms in order to diagnose constipation, which may underestimate the true incidence of constipation: among PD patients, diagnosing constipation based on the objective measure of prolonged colorectal transit time leads to higher incidence than diagnosis based on subjective symptoms (3, 22). Future studies should verify and extend our findings with larger samples, preferably using a longitudinal design and combining subjective and objective criteria to diagnose constipation.

## Conclusion

Our study suggests that patients with MSA are prone to constipation, which more often precedes motor symptoms. MSA-P subtype and total UMSARS score may be independent risk factors for constipation in MSA. Our results may help guide future research to clarify MSA pathogenesis in its earliest stages.

## Data availability statement

The original contributions presented in the study are included in the article/supplementary material, further inquiries can be directed to the corresponding authors.

## Ethics statement

The studies involving human participants were reviewed and approved by the Ethics Committee of West China Hospital of Sichuan University. The patients/participants provided their written informed consent to participate in this study.

## Author contributions

YC collected the data, conducted the statistical analysis, interpreted the data, and writing the manuscript. HH and PZ collected the data. YX and YC revised the manuscript. All authors contributed to the article and approved the submitted version.

## Funding

This work was sponsored by Natural Science Foundation of Chongqing, China (Grant No. cstc2020jcyj-bshX0055).

## Conflict of interest

The authors declare that the research was conducted in the absence of any commercial or financial relationships that could be construed as a potential conflict of interest.

## Publisher’s note

All claims expressed in this article are solely those of the authors and do not necessarily represent those of their affiliated organizations, or those of the publisher, the editors and the reviewers. Any product that may be evaluated in this article, or claim that may be made by its manufacturer, is not guaranteed or endorsed by the publisher.
